# Synthesis, Spectroscopic Study and Radical Scavenging Activity of Kaempferol Derivatives: Enhanced Water Solubility and Antioxidant Activity

**DOI:** 10.3390/ijms20040975

**Published:** 2019-02-23

**Authors:** Sui-Ping Deng, Yi-Li Yang, Xing-Xing Cheng, Wen-Rong Li, Ji-Ye Cai

**Affiliations:** 1 Department of Chemistry, Jinan University, Guangzhou 510632, China; yyl0420@stu2016.jnu.edu.cn (Y.-L.Y.); 17817717548@stu2016.jnu.edu.cn (X.-X.C.); 18312736162@163.com (W.-R.L.); tjycai@jnu.edu.cn (J.-Y.C.); 2 State Key Laboratory of Quality Research in Chinese Medicines, Macau University of Science and Technology, Macau 000853, China

**Keywords:** kaempferol, gallium complex, water solubility, antioxidant activity, flavonoid

## Abstract

Kaempferol (Kae) is a natural flavonoid with potent antioxidant activity, but its therapeutic use is limited by its low aqueous solubility. Here, a series of Kae derivatives were synthesized to improve Kae dissolution property in water and antioxidant activity. These compounds included sulfonated Kae (Kae-SO_3_), gallium (Ga) complexes with Kae (Kae-Ga) and Kae-SO_3_ (Kae-SO_3_-Ga). The compound structures were characterized by high-resolution mass spectrometry (HRMS), nuclear magnetic resonance (NMR) spectroscopy, ultraviolet-visible (UV-Vis) spectroscopy, Fourier transform infrared (FT-IR) spectroscopy and thermal methods (TG/DSC). The results showed that a sulfonic group (-SO_3_) was successfully tethered on the C3’ of Kae to form Kae-SO_3_. And in the metal complexation, 4-CO and 3-OH of the ligand participated in the coordination with Ga(III). The metal-to-ligand ratio 1:2 was suggested for both complexes. Interestingly, Kae-SO_3_-Ga was obviously superior to other compounds in terms of overcoming the poor water-solubility of free Kae, and the solubility of Kae-SO_3_-Ga was about 300-fold higher than that of Kae-Ga. Furthermore, the evaluation of antioxidant activities in vitro was carried out for Kae derivatives by using α,α-diphenyl-β-picrylhydrazyl (DPPH) and 2,2’-azino-bis(3-ethylbenzo-thiazoline-6-sulfonic acid) diammonium salt (ABTS) free radical scavenging. The results showed that Kae-SO_3_-Ga was also optimal for scavenging free radicals in a dose-dependent manner. These data demonstrate that sulfonate kaempferol-gallium complex has a promising future as a potential antioxidant and as a potential therapeutic agent for further biomedical studies.

## 1. Introduction

In general, the most discussed feature of polyphenols and plant phenolics is their capability to scavenge reactive oxygen species (ROS) [1,2,3]. However, the phenolics cannot be synthesized by human beings, but can be gained through the diet. Vegetables and fruits are the major indispensable sources of dietary phenolics [4,5]. Excess free radicals generated in body metabolism can be sufficiently decreased by regular intake of enough vegetables and fruits [6]. 

Flavonoids, which are phenolic compounds, extensively spread in the plant kingdom. The four major types of flavonoids include 4-oxoflavonoids (flavones and flavonols), isoflavones, flavan-3-ol derivatives (catechin and tannins) and anthocyanins [7]. Flavonoids are composed of two aromatic rings (rings A and B) linked through an oxygenated heterocycle (ring C). This ring C is the most representative core feature of each flavonoid subfamily [8]. 

Flavonoids exert a wide range of benefits to human health [9,10]. They are considered as one of irreplaceable antioxidants in our daily diet because they function as singlet oxygen scavengers, free radical quencher, or chelators of metal ions, which catalyze the oxidative reactions [11]. It was suggested that free radicals and lipid peroxidation were involved in several human diseases and age-related pathologies [12]. Flavonoids can directly scavenge superoxide and peroxynitrite in an effective way, block the reaction of free radicals in the nitric oxide signaling pathway in different classes of cells [13], and hinder injury caused by free radicals. 

Interestingly, the antioxidant activity of flavonoids is affected by the number of their hydroxyl groups. The performance of propyl gallate and other gallates with longer chain containing polyhydroxy groups is more preferable than the monohydric antioxidants. However, the antioxidant activity starts to go down to a lower level in structures having more than three hydroxy groups [14]. Moreover, the poor water solubility of flavonoids restricted their use. Hence, new strategies need to be developed to improve the activities of polyhydroxy flavonoids. 

Flavonoids are ideal antioxidants not only due to their radical scavenging ability but also their power to chelate with metal ions [15,16,17]. Their metal complexes are usually found to be more active when compared with free ligands [18]. For instance, Jabeen et al. [15] studied the antioxidant activity of Cu^2+^ and Fe^3+^ complexes with three flavonoids (morin, quercetin and 5-hydroxyflavone) by using ultralviolet-visible (UV-Vis) spectroscopy and cyclic voltammetry. The results showed these complexes were much better antioxidants than corresponding parent flavonoids. 

During the past few decades, the development of flavonoid-metal chelation has aroused a lot of interest, and continual efforts have been directed in synthesizing and characterizing novel flavonoid-metal ion complexes because of their potential applications in various fields [19]. Ravishankar et al. [20] synthesized ruthenium-conjugated chrysin analogues, which exactly displayed a 4-fold inhibition of platelet function and thrombus formation in vitro than chrysin. Medina et al. [21] synthesized apigenin-oxidovanadium compound and the complex showed moderate anti-cancer activity on lung A549 and cervix HeLa cell lines. 

Gallium (Ga) demonstrated potential biomedical applications due to its interesting physical and chemical properties. Ga was reactive in electron transfer process when it was placed in electrochemical environments. Evaluation of redox reaction between gallium-indium-stannum alloy and redox active species showed that Ga was more easily oxidized by losing electrons than other metals [22]. When Ga was coordinated with a redox-active ligand, it was able to regulate the electronic nature of the ligand [23]. ^67^Ga (half life 78 h) and ^68^Ga (half life 68 min) has been successfully used as imaging agents to assess certain types of tumors, inflammation, and other lesions [24,25,26,27]. 

**Figure 1:**
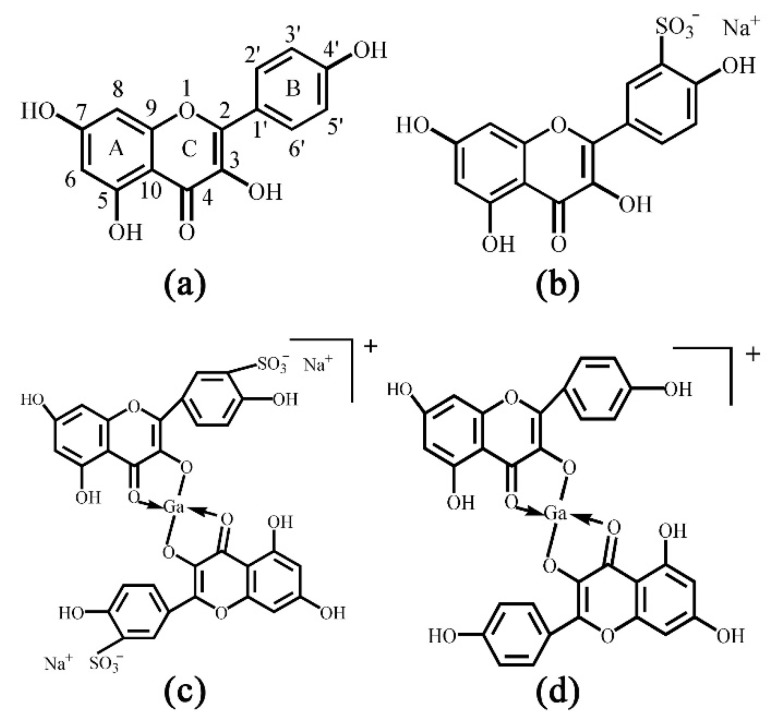
Chemical structures of (**a**) kaempferol (Kae): yellow powder, M_r_ 286.23, (**b**) sulfonated Kae (Kae-SO_3_): light yellow snowflake solid, M_r_ 365.29, (**c**) gallium (Ga(III)) complex with Kae-SO_3_ (Kae-SO_3_-Ga): brownish solid, M_r_ 798.28 and (**d**) Ga(III) complex with Kae (Kae-Ga): dark brown solid, M_r_ 640.17. The relative molecular weight without consideration of counter ions.

Kaempferol (Kae), well known for its prominent antioxidative activity [28], is a typical natural flavonol (Figure 1a). It is distributed in 80% of plant-based foods, including strawberries, grapes, apples, beans, kale, leek, broccoli, tomato, cabbage and tea, etc. [29]. It has been reported to have antioxidative, anti-microbial, anti-inflammatory, anti-diabetic and anti-cancer activities, as well as other pharmacologic activities against atherosclerosis and hyperlipidemia [30,31,32]. However, neither structural analyses nor biological activities of Ga(III) complex with Kae (Kae-Ga) have been described in detail. In this work, Kae was sulfonated in order to improve its water solubility. Besides, the coordination sites of Kae and sulfonated kaempferol (Kae-SO_3_) with Ga(III) cation were confirmed by UV-Vis spectroscopy, Fourier transform infrared (FT-IR) spectroscopy. The complexes were characterized by ^1^H and ^13^C nuclear magnetic resonance (NMR) spectroscopies, mass spectrometry and thermal methods. Moreover, 2,2-diphenyl-1-picrylhydrazyl (DPPH) and 2,2’-azino-bis (3-ethylbenzo-thiazoline-6-sulfonic acid) diammonium salt (ABTS) assays were used to evaluate antioxidant activities of the Kae derivatives.

## 2. Results and Discussion

### 2.1. Sulfonation of Kae on Ring B

Kae was sulfonated in order to improve its water-solubility. After sulfonation reaction, ^13^C and ^1^H NMR spectra of Kae and Kae-SO_3_ were obtained in DMSO-*d*_6_ solvent, respectively. In the NMR studies, all the protons and carbons resonate at their expected frequency ranges in DMSO-*d*_6_ at room temperature, properly assigned by the help of corresponding NMR experiments. A possible sulfonation site at Kae could be found by comparing the chemical shifts of carbon atoms in Table 1. The results indicated that the chemical shift of C3’ of Kae and Kae-SO_3_ was 115.91 ppm and 127.63 ppm, respectively. It showed the largest difference of about 12 ppm between Kae and Kae-SO_3_, and it might indicate that the chemical environment of C3’ changed greatly after Kae sulfonation. In addition, chemical shifts of other carbon atoms on ring B was also changed, but to a less extent about 2 ppm. Because the sulfonic group was a strong electron withdrawing group, it could affect the electron cloud density around the carbon atoms on ring B. However, the chemical shift of carbon atoms on ring A was nearly unchanged. Therefore, a sulfonic group was expected to replace the hydrogen atom of C3’ (Figure 1b). 

Furthermore, there was a lack of a signal of H_3’_ proton in ^1^H NMR spectra of Kae-SO_3_ and Ga(III) complex with Kae-SO_3_ (Kae-SO_3_-Ga) in Table 2, respectively. It proved the substitution of H_3’_ proton by a sulfonic group in ring B of Kae molecule. Meanwhile, chemical shifts of H_2’_ and 4’-OH protons also changed a little after the sulfonation reaction. Because the sulfonic group was a strong electron withdrawing group, it influences electronic cloud density of nearby hydrogen atoms.

### 2.2. UV-Vis Spectra Analysis

**Figure 2:**
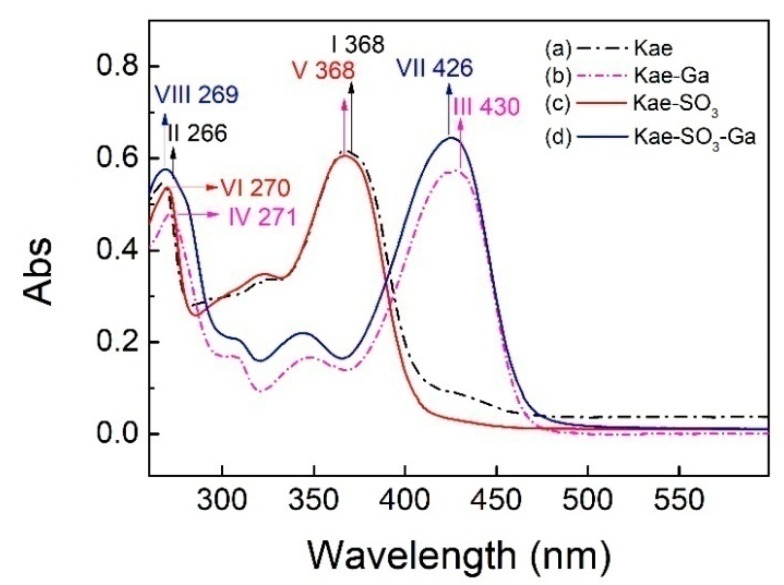
Ultraviolet-visible (UV-Vis) spectra of (**a**) Kae, (**b**) Kae-Ga, (**c**) Kae-SO_3_ and (**d**) Kae-SO_3_-Ga, respectively.

The UV–Vis spectra of Kae, Kae-SO_3_, Kae-Ga and Kae-SO_3_-Ga in ethanol were analyzed, respectively (Figure 2). The data is listed in Table 3. It was obvious that the corresponding profiles for Kae and Kae-SO_3_ were similar in Figure 2. The same pattern was also observed when comparing with the profiles for Kae-Ga and Kae-SO_3_-Ga. This UV pattern similarity supported the idea that -SO_3_ group has little effect on Kae’s UV absorbtion, and the chelation ability of Kae with Ga(III) was hardly affected by introducing a sulfonic functional group in the basic flavonoid skeleton. 

For Kae, two major characteristic bands, i.e. two absorption peaks, were observed at 368 nm and 266 nm for band I and band II, respectively (Figure 2a), which was consistent with the previous study [33]. The absorption peak at 266 nm (band II) was considered to be annotated for the absorption involving benzoyl system (ring A). The absorption peak at 368 nm (band I) was closely related to the cinnamoyl system (ring B) of Kae. After Kae chelating with a Ga(III) ion, band II moved slightly from 266 nm to 271 nm (band IV) by red shift, which suggested that no considerable change arose in benzoyl system in the chelating reaction. However, the interaction between Kae and Ga(III) caused a significant change to the peak at 368 nm (band I). It was displaced by the peaks at ~430 nm of Kae-Ga (band III) and Kae-SO_3_-Ga (band VII), respectively (Figure 2). A bathochromic shift of about 62 nm was clearly appeared. It confirmed that complex formation took place between Ga(III) ion and Kae ligand. 

Moreover, according to the Kae structure (Figure 1a), it could be inferred that the 3-hydroxy-4-oxo part of Kae might be the initial sites involved in the metal coordination process due to the acidic nature of 3-OH and appropriate location of 4-CO. It was well known that high delocalization of oxygen electrons of 3-OH group could prompt the π electrons delocalization [17,34]. After 3-OH group binding with a Ga(III) ion, a big outstretched π bond system could be developed subsequently via electronic redistribution. Thus, an enhanced conjugative effect occurred in association with 3-OH and 4-CO in ring C [35], and it brought a direct consequence—a new ring was formed in the Ga(III) complexes with Kae-Ga and Kae-SO_3_-Ga (Figure 1c,d). This newly-formed ring could enable these complexes to obtain additional molecular stabilization of lower energy state. 

### 2.3. FT-IR Spectra Analysis

**Figure 3:**
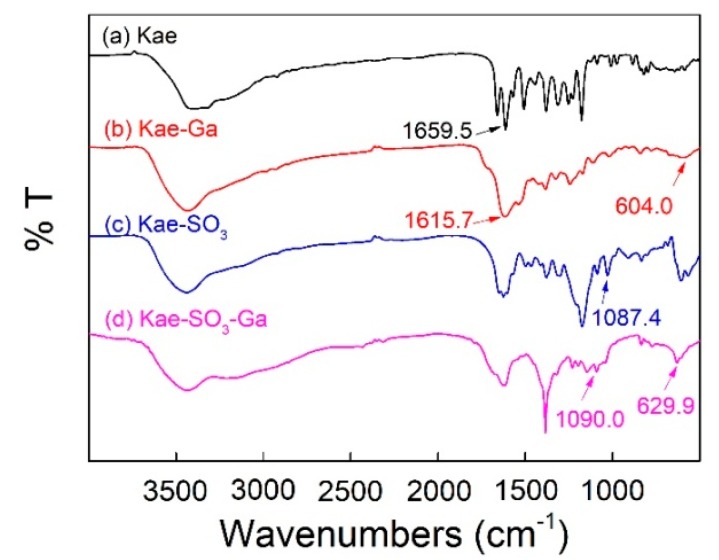
Fourier transform infrared (FT-IR) spectra of (**a**) Kae, (**b**) Kae-Ga, (**c**) Kae-SO_3_ and (**d**) Kae-SO_3_-Ga, respectively.

Figure 3 showed FT-IR spectra of Kae, Kae-Ga, Kae-SO_3_ and Kae-SO_3_-Ga, respectively. Data is listed in Table 4. For free Kae, the characteristic C=O stretching band was found at 1659.5 cm^−1^. It shifted to 1615.7 cm^−1^ and 1617.6 cm^−1^ in the case of Kae-Ga and Kae-SO_3_-Ga, respectively. This was because the metal coordination had a significant impact on 4-CO carbonyl group of ring C, and it confirmed the Ga(III) had bonded to 4-CO. In the spectra of Kae-Ga and Kae-SO_3_-Ga, the presence of unique vibration at 604.0 cm^−1^ and 629.9 cm^−1^, respectively, represented a Ga-O stretching band, which also indicated the formation of metal complex. However, free Kae and its sulfonate exhibited no such band. What is more, a slight increase in vibrational frequency of ѵ(C–OH) between Kae and Kae-Ga (1380.1 cm^−1^ to 1384.8 cm^−1^), as well as that between Kae-SO_3_ and Kae-SO_3_-Ga (1378.9 cm^−1^ to 1384.4 cm^−1^), suggested the involving of ѵ(C–OH) vibration of phenolic hydroxyl, which revealed that the metal complexation almost had no impact on phenolic hydroxyl. It confirmed the Ga(III) had bonded to 3-OH of ring C. The above outcomes indicated that the Ga(III) ion probably coordinated with Kae and Kae-SO_3_ at 3-OH and 4-CO of ring C. The presence of peaks at 1087.4 cm^−1^ and 1090.0 cm^−1^ belonged to S=O deformation band in the spectra of Kae-SO_3_ and Kae-SO_3_-Ga, respectively. This confirmed that -SO_3_ group had bonded to Kae. The broad ѵ(O–H) vibrational bands near 3400 cm^−1^ in all samples suggested the existence of water. 

### 2.4. High-Resolution Mass Spectrometry (HRMS) Analysis

**Figure 4:**
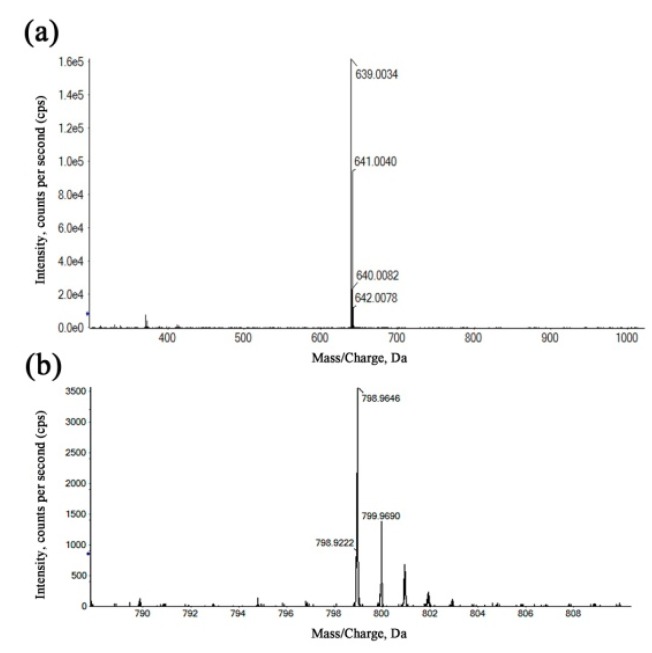
The high-resolution mass spectra (HRMS) of the kaempferol-gallium complexes. (**a**) 1:2 (metal-to-ligand ratio (M:L)) Kae-Ga, (**b**) 1:2 (M:L) Kae-SO_3_-Ga.

The refinement and upgrading of mass spectrometry techniques have given a considerable impetus to determine the composition, molecular weight distribution and structure of complicated materials [36]. In order to more accurately determine the structure of Ga(III) complexes, the coordination products were identified by HRMS using the positive ionization mode. In Figure 4a, the species with *m/z* = 639.0034 was assigned to a 1:2 (metal-to-ligand ratio (M:L)) complex for Kae-Ga, which was a consequence of the loss of two hydrogen atoms from two Kae molecules, and then chelating with one Ga(III) cation. In Figure 4b, the peak at *m/z* = 798.9646 represented two Kae-SO_3_ molecules and one Ga(III) ion, which also depicted a 1:2 (M:L) complex for Kae-SO_3_-Ga.

### 2.5. ^1^H NMR Spectrometry Analysis

Roy et al. [37] successfully synthesized a luteolin-vanadium(II) complex. The results showed that a vanadium(II) cation was complexed with 4-CO and 5-OH sites of luteolin. This hydroxyl chelation site was different from that in Kae-Ga and Kae-SO_3_-Ga, respectively. In the present work, however, the Ga(III)-binding sites on Kae were further confirmed by ^1^H NMR study (Table 2). Compared to the free Kae, the signal of 3-OH proton was absent in the metal complexes, including Kae-Ga and Kae-SO_3_-Ga (Table 2). However, the other three hydroxyl group protons (4’-OH, 5-OH and 7-OH) remained after chelation. This indicated that the Ga(III) ion combined with Kae through the 3-OH group. 

After the complexes were formed, ^1^H NMR data showed that chemical shifts of hydrogen atoms on the 3-OH had changed obviously, while those on the other hydroxyl groups changed slightly. It was probably attributed to the increase of the conjugation effect caused by the coordination when the complex was formed, and the subsequent increase of flavonoid planarity [30]. This result provided evidence that Kae successfully chelated with Ga(III) ion via 3-OH and 4-CO groups, and in the same way Kae-SO_3_ combined with Ga(III) ion. 

### 2.6. Thermal Study of the Kae-Ga Complex

**Figure 5:**
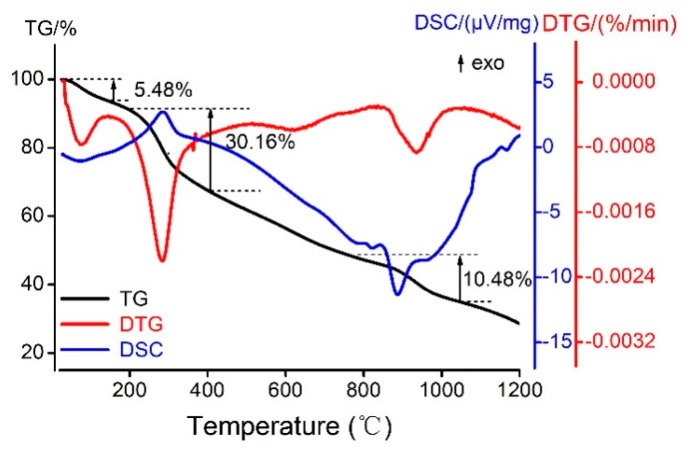
The thermogravimetric and differential scanning calorimeter (TG/DSC) curves of melting process of Kae-Ga complex in N_2_.

With the utilization of a simultaneous thermal analyzer, the Kae-Ga sample was detected under dynamic inert atmosphere (nitrogen) and the data of differential scanning calorimetry (DSC) and thermal gravity (TG) could be simultaneously obtained. Figure 5 showed the thermal analysis (TG/DSC) of Kae-Ga with the heating rate of 20 °C·min^−1^. TG and derivative thermogravimetric (DTG) plots showed that Kae-Ga exhibited a three-step degradation process. First, a slight weight loss (5.48%) was observed at 34–128 °C, and it was suggested that the complex contained two water molecules, which was 5.32% in calculation. Second, a significant weight loss (30.16%) could be seen at 297 °C, which denoted an exothermic peak. The Kae-Ga complex underwent decomposition, and was converted into carbon oxides and water. Third, in the DTG curve, the next peak in the temperature range of 844–1033 °C was related to the complete decomposition of the complex. The residue eventually turned out to be gallium oxide and remained stable. 

Through comprehensive analysis of the above three curves, the Kae-Ga complex exhibited a different degradation over temperatures of 800 °C when compared with other flavonoid-metal complexes in previous studies [38]. Kae-Ga complex decomposed finally over 900 °C, while other flavonoid complexes often below 800 °C. It might probably provide a clue that the Kae-Ga complex possessed a better stability in thermodynamics. 

### 2.7. Enhanced Water-Solubility of Kae-SO_3_ and Its Complex

Though Kae has many pharmacological activities, poor water solubility and dissolution of Kae has led to its low bioavailability, and subsequently few clinical applications. Certain techniques were thus used in previous studies to improve the aqueous solubility of Kae, such as nanoparticle and liposome delivery systems. Nanoparticle systems recently became a effective technique to significantly enhance dissolution and bioavailability of some poorly water-soluble drugs by reducing the size of the compounds from micron- to nanoscale, usually between 10 nm to 1000 nm. Lin et al. [39] demonstrated that the dissolution percentage of Kae nanoparticle (KAEN) (about 88 nm) was 139-fold higher than that of the pure Kae (about 6.2 μm) in a pH 1.2 hydrochloric acid buffer solution, and KAEN showed better antioxidant activity than Kae in water. Dissolution enhancement was related to the reduced particle size, high encapsulation efficiency and crystal-to-amorphous transformation of Kae, as well as hydrogen bonding between Kae with Eudragit E100 (EE100) and polyvinyl alcohol (PVA) as nontoxic excipients [39]. 

In addition, β-cyclodextrin (β-CD) and sulfobutylether β-CD (SBE-β-CD) were useful in overcoming poor water solubility of the flavonoids by inclusion complexation with the flavonoids in their hydrophobic holes, such as has been shown for Kae, luteolin and myricetin [40]. β-CD has a hydrophilic outer suface, and sulfobutylether is a negatively charged group. These factors led to understand that SBE-β-CD was more effective in increasing the water solubility and antioxidant activity of the flavonoids when compared with β-CD. Jung et al. [40] reported that solubility of the flavonoids with SBE-β-CD was about 10- to 50-fold higher than that of flavonoids without CD. 

Moreover, flavonoid sulphation or sulfonation was also an effective strategy to improve poor aqueous solubility of flavonoids by increasing flavonoid polarity. Sulphated flavonoids, such as persicarin and quercetin 3-sulphate, were often found in some specific plants. The flavonoids converted to their sulphate esters by enzymatic reactions in plant metabolism [41]. These substituent sulphate groups were negatively charged, which indicated that sulphated flavonoids had increasing polarity and water-solubility than their parent flavonoids. In fact, sulphated flavonoids showed several pharmacological effects, such as anti-inflammatory, anticoagulant and antitumor activities [41]. 

In the present study, however, sulfonated Kae and its complexes were successfully synthesized in vitro using a simple and quick preparation process. The solubility of kaempferol-based compounds in water at 30 °C is listed in Table 5. The aqueous solubility of free Kae and Kae-Ga was 3.95 × 10^−4^ mol·L^−1^ (0.0113 g/100 g soln.) and 6.22 × 10^−4^ mol·L^−1^ (0.0398 g/100 g soln.), respectively. The results proved the poor water solubility of free Kae and Kae-Ga, and these solubilities were of the same order of magnitude. However, the solubility of Kae-SO_3_ was obviously elevated to 6.42 × 10^−2^ mol·L^−1^ (2.49 g/100 g soln.). It was almost enhanced 220-fold when compared with that of Kae. Moreover, the solubility of Kae-SO_3_-Ga was significantly increased to 1.70 × 10^−1^ mol·L^−1^ (14.34 g/100 g soln.), which was almost enhanced 360-fold when compared with that of Kae-Ga. Furthermore, Kae-SO_3_-Ga had the highest solubility among the four compounds. The change of solubility showed that the solubility of Kae increased after transforming to its sulfonate, and a coordinated Ga(III) moiety to Kae-SO_3_ could greatly enhance water solubility further. Aromatic sulfonation has been recognized as an electrophilic substitution reaction. Sulfonate is prized for its ability to be easily soluble in water [42]. The sulfonic acid group is negatively-charged; therefore, this negatively-charged group could offer an increasing molecular polarity for Kae. The fact that Kae-SO_3_ was more polar than Kae allowed Kae-SO_3_ to obtain greater water solubility and dissolution. Besides, the coordinated trivalent Ga(III) imparted the complex inner part to a mono-charged ion; hence, the complex inner part still maintained polarity. In addition, Ga(III) might tune the electronic nature of the ligand to assist in improving water solubility. Thus, it could be inferred that solubility improvement of poor water-soluble flavonoids could be achieved by sulfonation of flavonoids, followed by metal complexation.

### 2.8. Antioxidant Activities of Kae, Kae-SO_3_ and Their Complexes

The metal ion complexation with the flavonoids may affect the chemical properties of flavonoid molecules, and therefore lead to changes in the antioxidant activities. Roy et al. [37] reported that both luteolin and luteolin-vanadium(II) complex showed antioxidant activities in a time- and dose-dependent manner by using DPPH, ABTS and ferric reducing antioxidant power (FRAP) methods. However, luteolin-vanadium(II) complex showed better antioxidant activity than luteolin. The compounds could donate electron or hydrogen atom, and react with free radicals or terminate the chain reaction. 

In the present study, the results were in agreement with the previous findings that Ga(III) complexation affected the antioxidant activity of the ligands. There is not a universal method to measure antioxidant activity. Hence, two different measurements have been used to assess the antioxidant activity of Kae-based compounds. 

**Figure 6:**
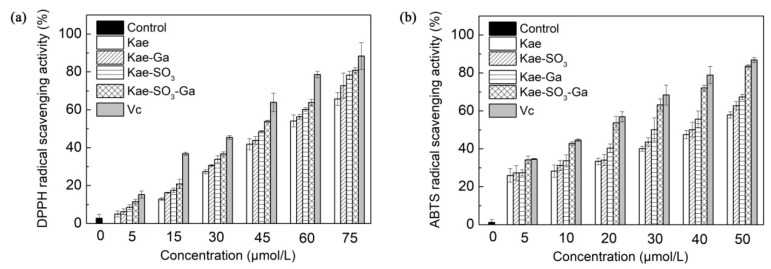
Effects of Kae-based compounds on (**a**) α,α-diphenyl-β-picrylhydrazyl (DPPH) radical scavenging activity and (**b**) 2,2’-azino-bis (3-ethylbenzo-thiazoline-6-sulfonic acid) diammonium salt (ABTS) radical scavenging activity. L-Ascorbic acid (Vc) as standard radical scavenger. Water-soluble Kae-SO_3_, Kae-SO_3_-Ga and Vc were dissolved in phosphate buffered saline (PBS) solution, respectively, while others in ethanol. Data was expressed as means ± SD obtained in three independent experiments. Significant difference was found from Kae derivative groups and Vc group to the control (*p* < 0.05).

DPPH, a stable free radical, is used widely to assess the antioxidant capacity of hydrogen donors or free radical scavengers such as plant extracts and food matrix [43]. In the radical scavenging reaction, antioxidants donate a hydrogen radical or an electron to DPPH^·^ and transform it into a diamagnetic molecule; thus, the antioxidative activity primarily relies on hydrogen- or electron-donating capacity. According to previous studies, Kae was a flavonoid well known for its good radical scavenging activity and antioxidant activity [44]. Therefore, DPPH radical scavenging activities of Kae, Kae-Ga, Kae-SO_3_, Kae-SO_3_-Ga and L-Ascorbic acid (Vc) with different concentrations were measured, respectively. Water-soluble Kae-SO_3_, Kae-SO_3_-Ga and Vc were dissolved in phosphate buffered saline (PBS) solution, while water-insoluble Kae and Kae-Ga in ethanol. The results are shown in Figure 6a and Table 6a. 

It was observed that DPPH radical scavenging activity was increased significantly for all compounds with increase of their concentrations in the range of 0–75 μmol·L^−1^ when compared with the control (*p* < 0.05). It was attributed to the scavenging ability of the Kae, Kae-Ga, Kae-SO_3_, Kae-SO_3_-Ga and Vc as standard radical scavenger. At the concentration of 75 μmol·L^−1^, Kae showed about 66% radical scavenging activity towards DPPH, while Kae-SO_3_-Ga about 82%, the highest value than other three Kae-based compounds. Thus, the results indicated that Kae-SO_3_-Ga had the best radical scavenging activity, and these modifications in Kae appeared to offer a workable way to improve the free radical scavenging activity. 

In order to confirm the radical scavenging activity, the absorption of active ABTS assay at 734 nm in the presence of these Kae-based compounds was observed. Figure 6b showed the ABTS^•+^ radical scavenging abilities of Kae-based compounds at different concentrations, respectively. Compared with the DPPH radical scavenging ability, Kae-based compounds showed a higher ABTS^•+^ radical quenching capacity, which might be due to the different reaction mechanisms of the used methods [45]. Among the Kae-based compounds, Kae-SO_3_-Ga was the most effective compound for scavenging ABTS^•+^ radical, which was almost closed to Vc, the standard material. For instance, at a concentration of 50 μmol·L^−1^, Kae scavenged free radical about 58%, while Kae-SO_3_-Ga about 84%, the highest value than other three Kae-based compounds (Table 6b). 

The distinct antioxidant activity of Kae-SO_3_-Ga could be due to its obviously improving water-solubility and enhancing electron-donating capacity through a chelating Ga(III) ion with ring C. Moreover, the active hydroxyl groups of Kae were retained in Kae-SO_3_ and Kae-SO_3_-Ga after sulfonation. Thus, Kae-SO_3_-Ga obtained an enhancing ability to stabilize unpaired electrons and then scavenge free radicals. It was thus suggested that Kae-SO_3_-Ga was more suitable as an antioxidant than the parent Kae due to its potential to scavenge free radicals more effectively. 

It is well known that flavonoids have many beneficial pharmacological and biological functions, such as antioxidant, anticoagulant, immunomodulatory, anti-inflammatory, anti-microbial, etc. [41,46]. However, some types of flavonoids showed cytotoxicity against normal healthy cells. Wangchuk et al. [46] reported that luteolin exhibited considerable cytotoxicity on a human normal cholangiocyte cell line. Kae, quercetin and isoquercitrin showed hemolytic potential on human erythrocytes [47]. In our preliminary study about cytotoxicity, interestingly, Kae and its complexes showed low cytotoxicity against normal epithelial cells, but were reasonably toxic on breast cancer cells. Further studies would be carried out on their potential biological activities. 

## 3. Conclusions

Based on flavonoid Kae, three compounds including sulfonated Kae, the Ga(III) complexes with Kae and sulfonated Kae were successfully synthesized and characterized by means of UV-Vis and FT-IR spectroscopies, ^1^H- and ^13^C-NMR spectrometry, mass spectrometry and thermogravimetric analysis. Sulfonated Kae and sulfonated Kae-Ga(III) complex showed greater water-solubility than Kae, revealing that the poor water-solubility of flavonoids was overcome by sulfonation, which was related to the increasing molecular polarity by negatively-charged sulfonic group and the complex inner shell. The DPPH and ABTS radical scavenging activity by using UV–Vis spectroscopy under imitated physiological conditions revealed that Kae-SO_3_-Ga exhibited the best antioxidant activity than Kae, Kae-Ga and Kae-SO_3_. Both flavonoid sulfonation and metal ion have impacts on the antioxidative ability of free flavonoid, revealing that the sulfonated flavonoid-metal complex obtains optimal antioxidant activity through enhanced water-solubility and electron-donating capacity. It could be inferred that sulfonated flavonoid-metal complexes might be potential antioxidants for therapeutic studies. However, further studies are required to understand their fate in the biological system as well as their mechanisms of action in health and disease. 

## 4. Materials and Methods

### 4.1. Materials and Instrument

Kae (98%) was purchased from Meilun Biotechnology Co. Ltd. (Dalian, China). Gallium nitrate (Ga(NO)_3_), potassium persulfate (K_2_S_2_O_8_), DPPH and ABTS assays were delivered by Aladdin Biochemical Co. Ltd. (Shanghai, China). Ethanol was obtained from Tianjin Damao Chemical Reagent Factory (Tianjin, China). The stock solutions of Kae and Ga salt were prepared in ethanol.

The UV-Vis spectra were recorded by a Cary 5000 UV-Vis-NIR spectrometer (Varian, Palo Alto, CA, USA) with a wavelength range of 200–800 nm. The FT-IR spectra were recorded in the range of 400–4000 cm^−1^ using a Nicolet 6700 art visible spectrometer (Thermo Scientific, Madison, WI, USA) and samples were prepared in the solid state (KBr pellets). TG/DSC analysis of the complex was carried out by a thermal analyzer of TGA/DSC 3^+^ (Mettler Toledo, Schwerzenbach, Switzerland).

### 4.2. Synthesis of Kae Derivatives 

Kae-Ga: Kae-Ga was synthesized according to a previous study with appropriate modifications [48]. Kae and Ga(NO)_3_ were dissolved in ethanol. The two solutions were mixed and refluxed. The solvent was then removed under reduced pressure. The dark brown residues were washed three times with ethanol and dried in air, and its yield was 81.29%. 

Kae-SO_3_: Kae was added to concentrated sulfuric acid. The mixture was stirred at 60 °C, then dispersed into saturated sodium chloride solution and filtered to remove the solvent under reduced pressure. The filtered solids were dissolved in distilled water and appropriate sodium chloride was added to it. Flocculent deposits were found after a period of 12 h of static culture. Lastly, the precipitate was filtered and dried in air, presenting as light yellow snowflake microcrystalline and its yield was 73.12%. 

Kae-SO_3_-Ga: Kae-SO_3_ was dissolved in 20 mL distilled water, followed by introducing 10 mL aqueous solution of Ga(NO)_3_. The mixture was refluxed at 70 °C. The solid brownish precipitate was rinsed carefully by distilled water, then evaporated rotationally and dried in air, and its yield was 65.92%. 

### 4.3. UV-Vis Spectra, FT-IR Spectra and TG/DSC Measurements

UV-Vis spectra of Kae and its Ga(III) complexes were obtained by a double beam spectrophotometer. The dynamics of the development of the complexes and the interactions between Kae and Ga(III) ion were detected by monitoring the changes in UV-Vis spectrum. Infrared spectra were recorded in KBr pellets with a spectrometer in the range of 400–4000 cm^−1^. The TG/DSC analysis of the complex was carried out by a thermal analyzer of TGA/DSC 3^+^ at a heating rate of 20 °C min^−1^ between 25–1000 °C and under N_2_ atmosphere (flow rate: 10 mL·min^−1^). 

### 4.4. NMR Spectrometry

^1^H NMR and ^13^C NMR experiments were carried out by a Bruker Avance 300 MHz NMR spectrometer in DMSO-*d*_6_ using tetramethyl silane (TMS) as internal standard, and the chemical shift values of hydrogen atoms and carbon atoms were reported in parts per million (ppm), respectively.

### 4.5. HRMS

Positive ion mode of detection was employed in HRMS by using the X500R QTOF system (AB SCIEX, Framingham, MA, USA). Sample solutions were continually infused via a syringe pump at a flow rate of 0.3 μL·min^−1^. The results of mass spectrometry confirmed that the Kae metal complexes were successfully synthesized due to peaks at *m/z* = 639.0034 and 798.9646 for Kae-Ga and Kae-SO_3_-Ga, respectively. 

### 4.6. Measurements of Antioxidant Activity by DPPH and ABTS Radicals

The DPPH radical scavenging assay was used according to the previous method with some modifications [49]. Briefly, Kae and Kae-Ga were pre-dissolved in ethanol, while Kae-SO_3_ and Kae-SO_3_-Ga in PBS solution. Then different concentrations of Kae-based compounds (5, 15, 30, 45, 60, 75 × 10^−6^ mol·L^−1^) was added to DPPH solution (5 × 10^−5^ mol·L^−1^), respectively, at room temperature. The reaction was kept in the dark for 40 min. Then the absorbance of samples (A) at 517 nm was recorded and compared with that of control (A_0_), which was prepared in an analogous method with no addition of the Kae-based compounds. Vc was used as a standard radical scavenger for comparison. The suppression ratio for DPPH was calculated from the following expression: Scavenging ratio (%) = [(A_0_ − A)/A_0_] × 100%. 

The ABTS^•+^ radical scavenging activity of Kae-based compounds was measured using the previous method with some modifications [50]. The radical cation was prepared by mixing a 7.4 mmol·L^−1^ ABTS^•+^ stock solution with 2.6 mmol·L^−1^ K_2_S_2_O_8_ (1:1, *v*/*v*), and the mixture was incubated for 14 h in darkness at 4 °C. The ABTS^•+^ solution was diluted to the absorbance approximately 0.7 at 734 nm to gain an ABTS^•+^ working solution. Sample solutions (5, 10, 20, 30, 40, 50 × 10^−6^ mol·L^−1^) and ABTS^•+^ working solution were mixed together, respectively, and placed at room temperature for 5 min before detection. A solution without sample was set as a control. The absorbance of samples (A) and that of control (A_0_) was recorded at 734 nm. ABTS^•+^ scavenging activity was calculated by the following formula: Radical scavenging ratio (%) = (A_0_ − A/A_0_) × 100%.

### 4.7. Statistical Analysis 

All experiments were repeated at least three times. Data was analyzed using one-way analysis of variance (ANOVA) and Tukey’s test by SPSS software. *p* < 0.05 was considered significant. 

## Figures and Tables

**Table 1:** ^13^C nuclear magnetic resonance (NMR) chemical shifts (ppm) for Kae and Kae-sulfonate. The differences resulting from sulfonation *Δ* = *δ*(Kae-SO_3_) − *δ*(Kae) are also given below.

	Kae	Kae-SO_3_	*Δ*
C2	156.65	155.35	−1.3
C3	136.13	136.56	0.43
C4	176.38	176.42	0.04
C5	164.36	164.52	0.16
C6	98.67	98.76	0.09
C7	161.18	161.2	0.02
C8	93.95	93.9	−0.05
C9	147.29	146.36	−0.93
C10	103.52	103.55	0.03
C1’	122.14	122.01	−0.13
C2’	129.98	131.08	1.1
C3’	115.91	127.63	11.72
C4’	159.66	156.65	−3.01
C5’	115.91	117.32	1.41
C6’	129.98	129.18	−0.8

**Table 2:** Chemical shifts of hydrogen atoms in ^1^H NMR spectra for Kae, Kae-Ga, Kae-SO_3_ and Kae-SO_3_-Ga, respectively.

Compound	3-OH	7-OH	5-OH	4’-OH	H_6’_	H_2’_	H_5’_	H_3’_	H_8_	H_6_
Kae	12.49	10.80	10.12	9.42	8.06	8.03	6.95	6.92	6.44	6.19
Kae-Ga	—	10.32	10.23	7.92	7.80	7.77	6.84	6.81	6.55	6.36
Kae-SO_3_	12.50	10.99	10.89	9.61	8.06	8.42	6.96	—	6.46	6.20
Kae-SO_3_-Ga	—	11.04	8.55	8.08	7.79	7.40	7.24	—	7.07	6.87

**Table 3:** Ultraviolet-visible (UV-Vis) spectra data of Kae, Kae-Ga, Kae-SO_3_ and Kae-SO_3_-Ga, respectively.

Compound	Peak I (nm)	Peak II (nm)
Kae	368	266
Kae-Ga	430	271
Kae-SO_3_	368	270
Kae-SO_3_-Ga	426	269

**Table 4:** Frequencies of characteristic absorption bands in Fourier transform infrared (FT-IR) spectra of Kae, Kae-Ga, Kae-SO_3_ and Kae-SO_3_-Ga, respectively.

Compound	–OH	C=O	C=C	C–OH (Phenolic Hydroxyl)	C–O–C	O–M	–SO_3_Na
Kae	3386.1	1659.5	1507.3	1380.1	1176.4	—	—
Kae-Ga	3434.9	1615.7	1533.3	1384.8	1170.0	604.0	—
Kae-SO_3_	3438.9	1654.7	1502.2	1378.9	1174.2	—	1087.4
Kae-SO_3_-Ga	3438.9	1617.6	1513.9	1384.4	1197.2	629.9	1090.0

**Table 5:** The solubility of the Kae-based compounds in water at 30 °C.

Compound	Solubility (g/100 g soln.)	Concentration (mol·L^−1^)
Kae	0.0113 ± 0.0026	3.95 × 10^−^^4^
Kae-SO_3_	2.49 ± 0.20	6.42 × 10^−^^2^
Kae-Ga	0.0398 ± 0.0040	6.22 × 10^−^^4^
Kae-SO_3_-Ga	14.34 ± 0.34	1.70 × 10^−^^1^

**Table 6:** (**a**) DPPH free radical scavenging ratio of different concentrations of Kae-based compounds. (**b**) ABTS free radical scavenging ratio of different concentrations of Kae-based compounds.

**(a)**					
**Concentration (×10^−6^ mol·L^−1^)**	**Kae (%)**	**Kae-Ga (%)**	**Kae-SO_3_ (%)**	**Kae-SO_3_-Ga (%)**	**Vc (%)**
5	5.07	6.24	8.60	11.53	15.29
15	12.85	16.26	17.51	20.85	36.79
30	27.34	30.63	33.89	36.75	45.44
45	41.84	43.90	48.48	53.92	63.93
60	54.13	56.36	63.90	65.68	78.65
75	65.75	72.77	80.85	82.47	88.37
**(b)**					
**Concentration (×10^−6^ mol·L^−1^)**	**Kae (%)**	**Kae-Ga (%)**	**Kae-SO_3_ (%)**	**Kae-SO_3_-Ga (%)**	**Vc (%)**
5	5.07	6.24	8.60	11.53	15.29
15	12.85	16.26	17.51	20.85	36.79
30	27.34	30.63	33.89	36.75	45.44
45	41.84	43.90	48.48	53.92	63.93
60	54.13	56.36	63.90	65.68	78.65
75	65.75	72.77	80.85	82.47	88.37
